# Inter-Regional Patients’ Migration for Hospital Orthopedic Intensive Rehabilitation: The Italian Experience

**DOI:** 10.3390/ijerph192113726

**Published:** 2022-10-22

**Authors:** Giovanni Guarducci, Gabriele Messina, Simona Carbone, Andrea Urbani, Nicola Nante

**Affiliations:** 1 Post Graduate School of Public Health, University of Siena, 53100 Siena, Italy; 2 Department of Molecular and Developmental Medicine, University of Siena, 53100 Siena, Italy; 3 General Directorate for Health Planning, Ministry of Health, 01144 Rome, Italy

**Keywords:** patient’s mobility, Gandy’s Nomogram, intensive orthopedic rehabilitation, health policy, health services quality and financing, Italian National Health Service, Italian regions, GIS

## Abstract

Background: Following the introduction of administrative federalism in the Italian National Health Service, inter-regional patients’ mobility has become increasingly relevant because, in addition to being an indirect index of the quality of care, it has important economic and financial implications. This study aimed to evaluate the fulfillment of the need for hospital orthopedic intensive rehabilitation on site and care-seeking patients’ migration to other regions. Methods: From 2011 to 2019, the data of intensive orthopedic rehabilitation extracts from the Hospital Discharge Cards provided by Italian Ministry of Health were analyzed. We studied the hospital networks of every Italian region (catchment areas). The epidemiological flows of inter-regional mobility were analyzed with Gandy’s Nomogram, while the financial flows were analyzed through Attraction Absorption and Escape Production Indexes. Results: Gandy’s Nomogram showed that only Piedmont, Lombardy, A.P. of Trento, E. Romagna, Umbria and Abruzzo had good public hospital planning for intensive orthopedic rehabilitation, with a positive balance for all studied periods. Lombardy, E. Romagna, Piedmont, Veneto and Latium have absorbed approximately 70% of all financial flows (about EUR 60.5 million). Conclusions: Only six regions appear to be able to satisfy the care needs of their residents, with a positive epidemiological and financial balance for all studied periods.

## 1. Introduction

Willingness to travel from an area of residence to receive healthcare dates back centuries, and persists in modern times [1,2]. In fact, the growth in globalization, the development of new forms of political cooperation, technological evolution and refinement, and a growing international market for medical care are key factors for the rise in patients’ movement across international borders [3]. This is best exemplified in the European Union due to the Directive on cross-border migration, which has promoted the free movement of patients between member states [4,5,6]. In addition, the European Union actively supports its citizens, who seek cross-border healthcare, by providing information through brochures, fact sheets and specialized contact points [7]. This means that the study of this phenomenon has become common in several disciplines (public health, jurisprudence, economics, geography and sociology) [8,9]. Evidence of health mobility also exists within the member states such as Italy, the United Kingdom, Spain, Sweden, and Belgium [10,11,12]. In Italy, the National Health Services (NHS), established in 1978, states that Italian citizens, according to their needs (prevention, hospital, rehabilitation, etc.), are free to choose the provider and the place of care [13,14,15]. This right grew in importance at the beginning of the 1990s, when the process of regionalization (and managerialization) started, which has decentralized the NHS into 21 regional health care systems, creating a “quasi-market” model to improve the quality and efficiency of hospital services, with potential consequences on equal access [16,17,18]. In 2001, the health-care competencies switched from a national to a regional level, leading to regions becoming responsible for all the health-care needs of their residents [19]. Some regions, such as Lombardy, have encouraged this competitive system within the public sector and between public and accredited private hospitals. Alternatively, in other regions, such as E. Romagna, policymakers have favored cooperation and coordination among providers by centrally planning production capacity and promoting a strong integration between hospital- and district-level services [20]. Although the effects of competition on the quality of health services are still unclear [21,22,23,24], citizens’ decisions to choose a hospital outside their region burden regional budgets, with the risk of undermining their sustainability. In fact, regions that cannot provide a good quality of service will see their resources drained by payments made to other regions for the services used by their residents [25,26,27]. For these reasons, it is essential to analyze healthcare mobility since it is a fundamental tool for health policy and planning; it is an indirect indicator of perception of hospital quality (real/perceived) [28,29]. Patients’ mobility for hospital orthopedic intensive rehabilitation is a topic that has not yet been addressed in the literature. The aims of our study were: (i) to analyze how the Italian regions have pursued the fulfillment of needs for hospital orthopedic intensive rehabilitation hospital services on-site and inter-regional patients’ migration trends; (ii) and to assess the financial impact of this phenomenon on regional funds.

## 2. Materials and Methods

### 2.1. Data and Catchment Areas

Data were collected from the Hospital Discharge Cards (HDCs) database of the Italian Ministry of Health, from 2011 to 2019. We considered the hospitalization of Italian patients for intensive rehabilitation for the Major Diagnostic Category of Diseases of Musculoskeletal System and Connective Tissue (MDC 8). We excluded the hospitalization of patients residing in other states, and the admissions of Italian patients to a foreign hospital. In addition, the inter-regional mobility flows of every Italian region were analyzed from data of Residents (R), Attraction (A) and Escapes (E).

### 2.2. Gandy’s Nomogram and Vectorial Analysis

Gandy’s Nomogram (NdG) was used to process data of R, A and E for every Italian region. It graphically represents access to hospital services by residents and non-residents in the studied period [30,31]. It is a squared area with a side of 100 placed in a Cartesian plane:

- The X value indicates Residents (R) out of Residents (R) plus the Attractions (A):

X = R/(R + A) × 100.

- The Y value indicates Residents (R) out of Residents (R) plus the Escapes (E):

Y = R/(R + E) × 100.

From 100 to 0, along the X-axis, the attraction increases, while along the Y-axis the escape to other regions increases. The Cartesian plane can be divided into four squares by two lines parallel to the axis, which start at X = 0; Y = 50 and X = 50; Y = 0. The diagonal that starts from the O point (X = 0; Y = 0) and ends at the W point (X = 100; Y = 100) splits the plane into a lower section where the X value is larger than the Y value, in which there are more escapes than attraction, and an upper one with an opposite situation. The points on the diagonal have the same value either for escapes or attractions, which are null in the W point and maximum in the O point. The four quadrants describe different scenarios of escapes and attractions:

- In the upper left quadrant, the number of attractions higher than both for resident admissions and escapes; the latter are lower than resident admissions (E < R < A). This condition is typical of “market oriented” regions, which can receive more funds since their hospitals admit more patients from other regions than patients from their own region. The point (X = 0, Y = 100) identifies the paradoxical condition in which there are no escapes and the regional hospital networks admit only patients from other regions.
- The upper right quadrant is divided into two sections by a bisector. In the upper section (hemi-quadrant of quality), escapes are lower than attractions and resident admissions are higher than attractions (E < A < R). In the lower area, attractions are less than the escapes, but the residents admissions are higher than the escapes (A < E < R). In this quadrant, there are regions that satisfy (in a more or less appropriate way depending on their position) the healthcare needs of their residents.
- The lower left quadrant is diagonally divided into two sections. In the upper area, the attractions are higher than escapes and resident admissions (R < E < A), while in the lower area, residents admissions are the lowest, and escapes are higher than attractions (R < A < E).
- The lower right quadrant shows the regions in which resident admissions are lower than escapes and higher than attractions (A < R < E).

For every year and region, we calculated the values of the X and Y axes to identify a point. All points from 2011 to 2019 have been linked in order to obtain a vector [5,32]. For those regions whose trend followed the same direction over the years (monophasic trend), the resulting vector was provided by linking the first (2011) to the last (2019) point. For those regions whose trend followed the same direction until a given year, then continued following another direction (biphasic trend), two vectors were created by linking the first point (2011) to the point of the year of direction change (first vector), while the second linked the year of direction change to the last one (2019). For the regions with more than two trend changes, we used the same methodology by linking, starting from 2011, the various changes of direction up to 2019.

### 2.3. Financial Evaluation

From the fees established by the agreements between the national government and the regions for the compensation of inter-regional healthcare mobility [33], we calculated the financial balance (A–E) for each region from the following rates:

- Daily rate for ordinary regime: €246.89; days over the cut-off value €143.13;
- Rate per access in day hospital regime: €197.51; accesses over the cut-off value €118.51.

The cut-off values from 2011 to 2013 were set at 60 days, dropping to 40 days from 2014 to 2019.

To describe the financial mobility flows absorbed and generated by each region, we used the Attraction Absorption and Escape Production Indexes [29].

To indicate the percentage of euros gained by region Xi (with i = 1, …, 21), out of the total euros gained by all regions, we used the Attractions Absorption Index (AAI):

AAI Xi = A Xi/Tot. A × 100

where AAI Xi = Attractions Absorption Index of region Xi; A Xi = Gain of region Xi; Tot. A = Total gain of all regions.

To indicate the percentage of euros spent for their escape by region Xi (with i = 1, …, 21), out of the total euros spent for escapes by all regions, we used the Escape Production Index (EPI):

EPI Xi = E Xi/Tot. E × 100

where EPI Xi = escape Production Index of region Xi; E Xi = euro spent for escapes by region Xi; Tot. E = total euro spent for escape by all regions.

### 2.4. Cartograpic Map

To provide an immediate and graphical representation of the two indexes described, we used Quantum Gis software version 2.16.3 (Open Source Geospatial Foundation Project) [34] to generate cartographic maps. The stratification of colors was performed based on percentiles (≤10%; between 11% and 25%; between 26% and 50%; between 51% and 75%; between 76% and 90%; and ≥91%) [29]; thus, we obtained maps with six different color intensities.

### 2.5. Statistical Analysis

The Shapiro–Wilk test was used to evaluate the normality of distribution. The student’s *t* test was used to assess differences between two groups. Cuzick’s test was used for trend analysis. Spearman’s rank correlation was calculated between AAI and IPE. Tests were performed using STATA software SE/14.0 (StataCorp LLC, College Station, TX, USA) and were considered statistically significant at 95% (*p* < 0.05).

The Shapiro–Wilk test was used to evaluate the normality of distribution. The student’s *t* test was used to assess differences between two groups. Cuzick’s test was used for trend analysis. Spearman’s rank correlation was calculated between AAI and IPE. Tests were performed using STATA software SE/14.0 (StataCorp LLC, College Station, TX, USA) and were considered statistically significant at 95% (*p* < 0.05).

## 3. Results

Table 1 shows the admissions for ordinary regime (ORD), day hospital (DH) and the ORD/DH ratio, from 2011 to 2019, divided into residents and mobility, for hospital orthopedic intensive rehabilitation. In Italy, during the study period, hospitalizations (ORD + DH) increased from 140,048 to 151,562 admissions (*p* < 0.05). For the ORD regime, they increased both for residents and in mobility (*p* < 0.05), while they both decreased for DH (*p* < 0.05). As a result, ORD/DH ratios increased significantly (*p* < 0.05). The ORD/DH ratios varied significantly if the hospitalizations were provided to residents or in mobility (*p* < 0.05).

**Figure 1:**
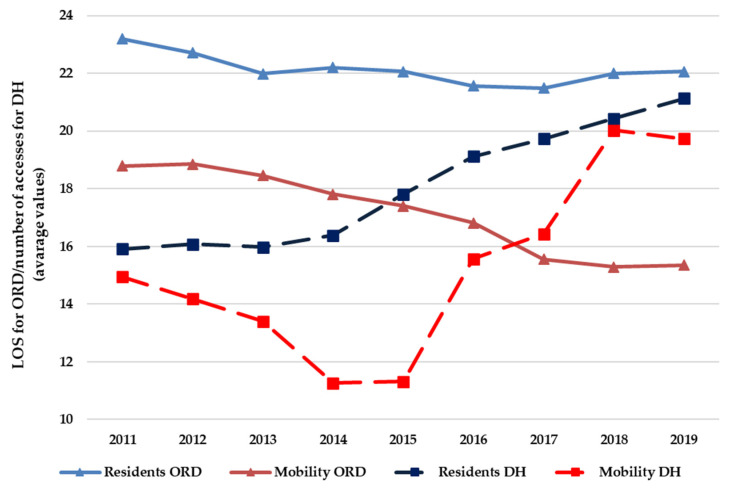
Average values of LOS for ORD and for the number of accesses for DH, for hospital orthopedic intensive rehabilitation (residents and mobility), from 2011 to 2019.

Figure 1 shows, for the hospital orthopedic intensive rehabilitation, the average length of stay (LOS) for ORD hospitalization and the number of accesses for each DH hospitalization, divided into residents and mobility, from 2011 to 2019. For the ORD regime, LOS decreased both for residents and for mobility admissions; for the latter, this decrease was significant (*p* < 0.05). For the DH regime, the number of accesses increased both for residents and for mobility admissions; for the former, this increase was significant (*p* < 0.05). The LOS for ORD and the number of accesses for DH were higher for residents than in mobility (*p* < 0.05).

**Figure 2:**
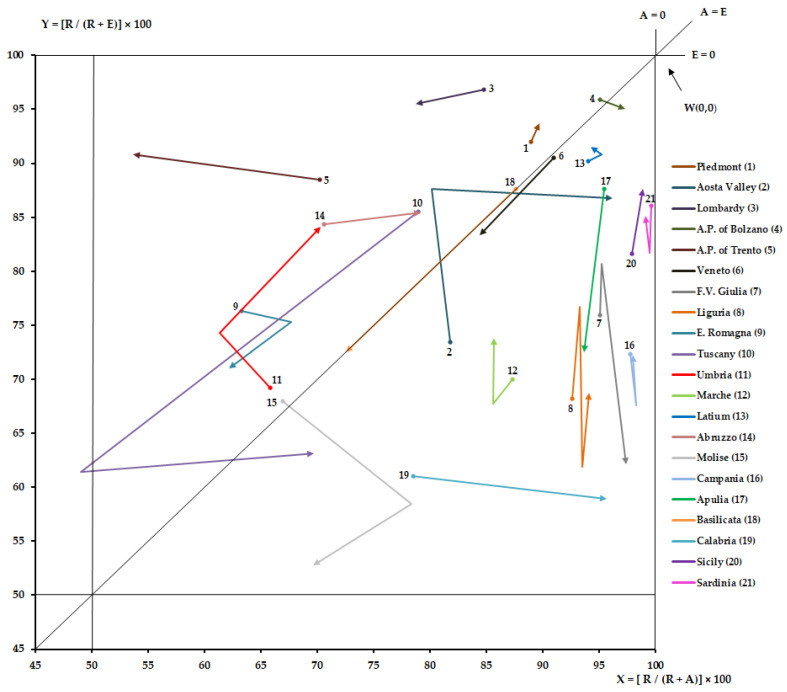
Zoom of NdG for hospital orthopedic intensive rehabilitation, from 2011 to 2019.

Figure 2 shows the NdG of every Italian region for hospital orthopedic intensive rehabilitation from 2011 to 2019. For the studied time periods, all regions were in the upper right quadrant. However, only six regions (Piedmont, Lombardy, A.P. of Trento, E. Romagna, Umbria and Abruzzo) were in the hemi-quadrant of quality. Tuscany appears in the upper left quadrant and then under the bisector in the upper right quadrant. A.P. of Bolzano and Molise lost their positions. Aosta Valley had initially crossed the bisector and then returned to the starting hemi-quadrant. Attractions increased (*p* < 0.05) for Lombardy, A.P. of Trento, Veneto and Basilicata, while they decreased (*p* < 0.05) for A.P. of Bolzano, Veneto, F.V. Giulia, Abruzzo, Calabria, and Sicily. Escapes increased (*p* < 0.05) for Veneto, F.V. Giulia, E. Romagna, Tuscany, Molise, Puglia and Basilicata, while they decreased (*p* < 0.05) for Piedmont, Aosta Valley, A.P. of Trento, Umbria, Abruzzo and Sicily.

**Figure 3:**
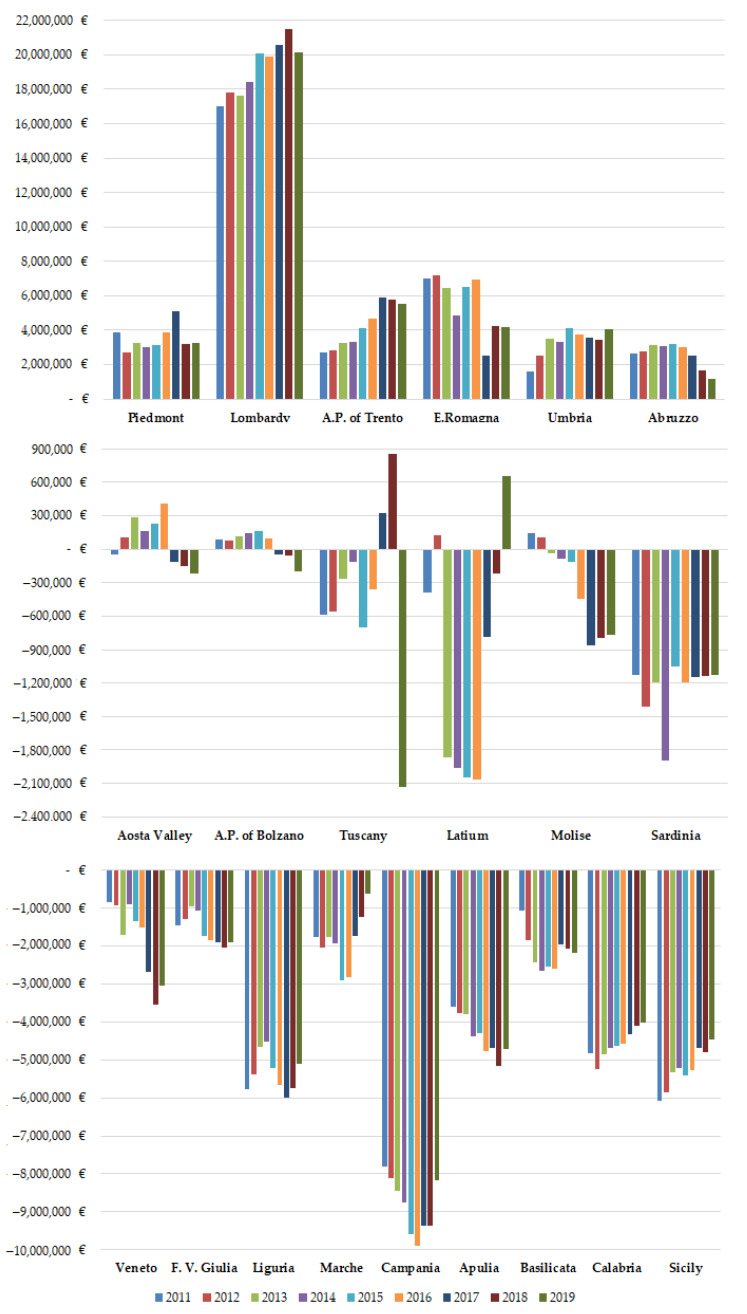
Financial mobility balance (A–F) of Italian regions for hospital orthopedic intensive rehabilitation from 2011 to 2019.

Figure 3 shows financial mobility balance (A–F) of all Italian regions, for hospital orthopedic intensive rehabilitation, from 2011 to 2019.

On average, the financial flows of mobility were EUR 86.7 ± 3.0 million. Only Piedmont, Lombardy, A.P. of Trento, E. Romagna, Umbria and Abruzzo had a positive balance for all studied periods. Aosta Valley, A.P. of Bolzano, Tuscany, Latium and Molise had positive balances for a few years. The other regions had constant negative balances. Only Lombardy (plus EUR 3.0 million) and A.P. of Trento (plus EUR 2.8 million) have increased their balance, and are already in surplus, significantly (*p* < 0.05). Veneto (minus EUR 2 million), F.V. Giulia (minus EUR 0.5 million), Molise (minus EUR 0.9 million) and Apulia (minus EUR 1.0 million) have worsened their negative balance (*p* < 0.05). E. Romagna (minus EUR 2.8 million) has decreased its earnings (*p* < 0.05). Calabria (plus EUR 0.8 million) and Sicily (plus EUR 1.6 million) tried to improve their negative balance (*p* < 0.05).

**Figure 4:**
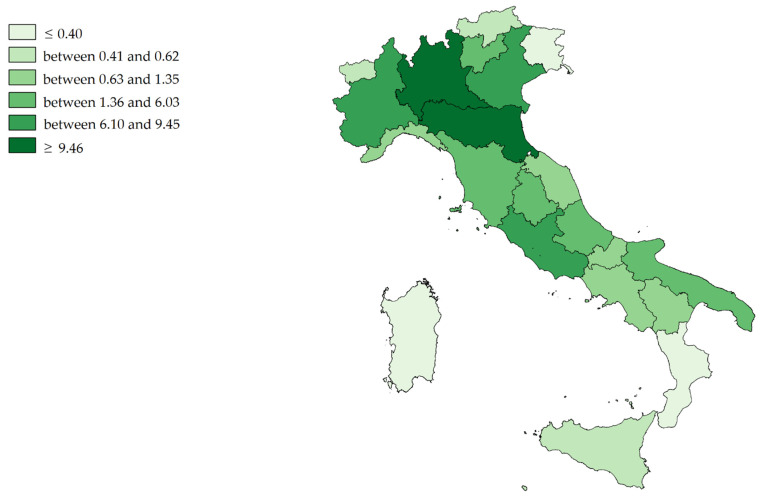
Cartographic representation of Attraction Absorption Indexes (AAI) for hospital orthopedic intensive rehabilitation, mean from 2011 to 2019.

Figure 4 shows the mean value (2011–2019) of the Attraction Absorption Indexes (A single region/Tot. A all regions) of all Italian regions for hospital orthopedic intensive rehabilitation. The hospital networks (dark green) of Lombardy, E. Romagna, Piedmont, Veneto and Latium have absorbed almost 70% (about EUR 60.5 million per year) of all financial flows. The gains of Sardinia, F.V. Giulia, Calabria A.P. of Bolzano, Aosta Valley and Sicily (light green) appear very slight (in total, about EUR 1.5 million per year).

**Figure 5:**
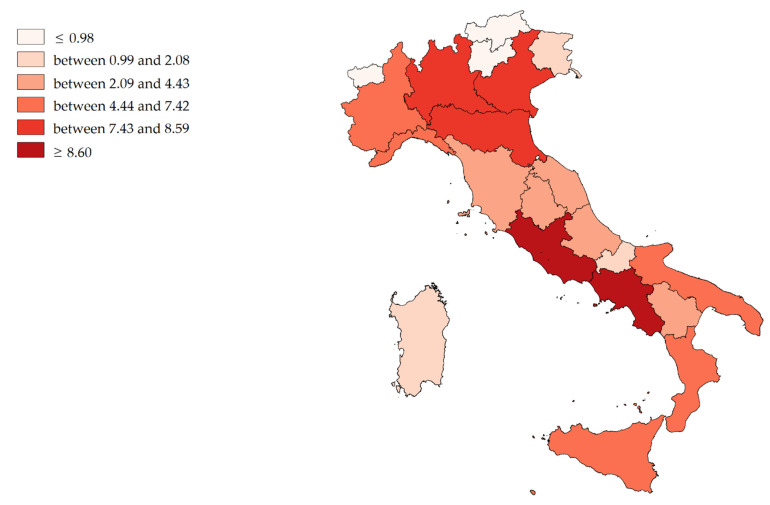
Cartographic representation of Escape Production Indexes (EPI) for hospital orthopedic intensive rehabilitation, mean from 2011 to 2019.

Figure 5 shows the mean value (2011–2019) of Escape Production Indexes (E single region/Tot. E all regions). Campania, Lombardy, Latium, Veneto and E. Romagna (dark red) have produced the largest financial outflows (in total, about EUR 39 million per year), while A.P. of Bolzano, Aosta Valley, A.P. of Trento, Molise, F.V. Giulia and Sardinia (light red) have produced the lowest (in total, about EUR 6 million per year).

Comparing Figure 4 and Figure 5, it can be seen that regions that have higher AAI Indexes also tend to have higher IPE Indexes (Spearman’s rho = 0.4896; *p* < 0.05), which is highlighted for the Northern regions (Spearman’s rho = 0.7667; *p* < 0.05).

## 4. Discussion

The topic of patients’ mobility is discussed at length both at the European level and in countries with regional healthcare systems [11]. At the Italian level, this phenomenon involves about 10% of hospital admissions. In our specific case (hospital intensive orthopedic rehabilitation), about 14% of patients moved to obtain healthcare, with a significantly increasing trend. Some healthcare mobility flows are due to the size of the catchment areas of high specializations, while others are due to the qualitative and quantitative insufficiency of the supply of care. This has an economic implication, including in the equity of the NHS. For the regions, there should be a priority to divide healthcare mobility for highly specialized care for low/medium complexity [27,29], to reduce the latter effect. Hospital orthopedic intensive rehabilitation falls within this category. Specifically, mapping flows by type of healthcare service is essential to identify the lack of supply so as to strengthen it in critical areas, guaranteeing equity of access to care and its quality [35]. The increase in orthopedic elective surgery and patients’ willingness to travel to receive this type of treatment is well known. The phenomenon of migration for hospital orthopedic intensive rehabilitation, resulting from acute hospitalization, has not yet been analyzed [36]. Because of this, the aim of our study was to analyze, from an epidemiological and financial point of view, the inter-regional mobility for hospital orthopedic intensive rehabilitation.

From 2011 to 2019, we found an increase in hospitalizations (+7.6%), both for residents and mobility, sustained only by admission in the ordinary regime (+10.6%). This may be due to the ever-increasing demand for orthopedic prosthesis surgeries, especially by older people [37,38], probably causing an increase in the ORD/DH ratio. In fact, according to the Italian rehabilitation guideline of the NHS, the DH is only provided when the patient’s clinical condition is stable, does not require 24 h nursing care and is able to tolerate daily home–hospital transfers [39]. Certainly, a patient’s mobility, regardless of clinical condition, will factor into the provision of the type of hospitalization, given the difference in ORD/DH ratios between residents and mobility patients. In fact, it is already known [40] that some hospitals, given their autonomy in patients’ admission, provide some care to patients from outside the area; in our case, an ORD admission instead of a DH was selected, to avoid daily transfers of the patient, which would be inconvenient for a patient coming from far away.

The rehabilitation guideline also recommends that the patient’s discharge and subsequent recourse to out-of-hospital care regimes should be performed when a condition of clinical stability is achieved, and highly complex diagnostic needs cease to exist [39]. In our case, the LOS for mobility admission decreased over the years and had a shorter duration compared to residents. Hospitals appear to have increased their efficiency in order to earn patients’ approval with their quick return home. This result contrasts with other studies [40,41,42], which show that patients who travel for care have a longer LOS. When patients must travel long distances, the criteria for discharge are likely to be stricter, resulting in more extended hospital stays. For example, there may be problems with travel time, pain control and the availability of medical facilities close to home [43].

For all studied periods, all regions were in the upper right quadrant of NdG, which indicates the ability to satisfy the care needs of their residents, as required by the NHS [44]. However, only Piedmont, Lombardy, A.P. of Trento, E. Romagna, Umbria and Abruzzo were in the upper “quality” part of the quadrant; these regions have a positive epidemiological balance (attraction is greater than escape). Tuscany, in 2018, seemed to have become increasingly attractive, outgrowing the hospitalization of its residents, but lost their position in 2019. Only A.P. of Trento has been able to reduce escapes and increase attractions at the same time, while Veneto and F.V. Giulia have decreased both their attractiveness and increased their escapes.

Hospital orthopedic intensive rehabilitation involves almost EUR 90 million, which is about 2% of all financial mobility flows [35]. In the studied period, and regions indicated in Gandy’s Nomogram, positive epidemiological balances also had positive financial balances. The case of Tuscany is interesting as the financial balances were negative in the first six years of the study unlike the epidemiological ones. The epidemiology, being the remuneration per day of hospitalization and not per single admission, masked the financial losses. Lombardy and A.P. of Trento have exploited this specialty to increase their gain. Veneto, F.V. Giulia, Molise and Apulia have worsened their already negative balances. E. Romagna decreased its already positive balances. Sicily and Calabria are trying to improve their financial situation. As found in another study [45], providers, to increase their income, might consider increasing their marketing for hospital orthopedic intensive rehabilitation.

According to AAI only five regions absorbed more than two-thirds of all financial mobility flows; Lombardy absorbed about one third, followed E. Romagna, Piedmont, Veneto and Latium. The regions producing the most outflows (EPI) were Campania, Lombardy, Latium, Veneto and E. Romagna.

In the north of Italy, regions with high income also had high expenditure. In the South, as observed in Campania, the picture is more heterogeneous, with regions absorbing little money but spending more. It is evident how the territorial dimension influences the phenomenon of the financial flows.

For regions with deficient balances, patients’ migration will have a doubly negative effect [29]. In addition to having to pay for their citizens’ hospitalization outside the region, if the escapes are due to a lack of quality, the regions will see their hospitals underused; however, they will still have to continue to fund them, according to the national laws. Moreover, if migration is due to a lack of supply, regions will still see some of the funds that could have been used to increase supply in their own territory drained away to other regions. These disparities, both in the lack of supply and its consequent equity of access and in the quality of care, may increase. For this reason, the central government should help regions redress healthcare inequalities [46].

### Limitations

Our study has the following limitations: The study is affected by a time gap of about 2.5 years. The current condition may be different from the one described, either due to programmatic interventions of various kinds that may have partially influenced trends and comparisons and/or due to being influenced by the COVID-19 pandemic that may have limited the patient’s migration. Despite this, the data analyzed are the most recent, and it was possible to profile trends over several years. The data of Italian HDCs have a long latency time; we analyzed only the latter since we consider them the most reliable. Our study does not quantify proximity mobility (movements from areas near regional borders), which are not related to improvements in the supply of healthcare, but due proximity and ease of access to hospitals in other areas. The small size of some regions and corresponding low population sizes could have an impact on the allocation of resources in health services.

## 5. Conclusions

The Italian situation appears heterogeneous: only six regions (four in the North, one in the Center, and one in the South) appear to be able to satisfy the care needs of their residents, with attractions minus escapes resulting in a positive financial balance.

It should be a priority for regions, especially those with negative financial balances, to reduce outgoing healthcare mobility for services such as hospital orthopedic intensive rehabilitation; outgoing healthcare mobility leads to financial losses and could undermine the equity of access to care for citizens.

The present study is a starting point for further investigation since patients’ mobility for hospital orthopedic intensive rehabilitation is a topic that has yet to be addressed in the literature. Our results can support decision-makers (politicians and managers) in finalized decision-making processes aimed at improving the supply (qualitative and/or quantitative) for hospital orthopedic intensive rehabilitation, given both the growing demand and the increasing of patients’ migrations.

## Figures and Tables

**Table 1:** Patients’ admission to Italian hospitals for hospital orthopedic intensive rehabilitation, 2011–2019.

Year	Residents			Mobility			Total			Total (ORD + DH)
	ORD	DH	ORD/DH	ORD	DH	ORD/DH	ORD	DH	ORD/DH	
2011	113,013	8892	12.71	17,674	469	37.68	130,687	9361	13.96	140,048
2012	114,272	7910	14.45	18,092	369	49.03	132,364	8279	15.99	140,643
2013	117,758	7655	15.38	19,188	324	59.22	136,946	7979	17.16	144,925
2014	116,282	7348	15.82	19,206	368	52.19	135,488	7716	17.56	143,204
2015	118,243	6271	18.86	20,955	346	60.56	139,198	6617	21.04	145,815
2016	124,117	6113	20.30	22,105	229	96.53	146,222	6342	23.06	152,564
2017	122,317	5917	20.67	22,704	195	116.43	145,021	6112	23.73	151,133
2018	122,523	5389	22.74	22,730	157	144.78	145,253	5546	26.19	150,799
2019	124,428	5239	23.75	21,736	159	136.70	146,164	5398	27.08	151,562

## Data Availability

Data obtained from HDCs database—General Directorate for Health Planning of Italian Ministry of Health upon specific request.
